# GIS Analysis of Alabama’s At-Risk Hospitals: Considering New Medicaid Changes and Potential Solutions

**DOI:** 10.1093/geroni/igaf122.3946

**Published:** 2025-12-31

**Authors:** Avani Shah, Joseph Swain

**Affiliations:** University of Alabama, Tuscaloosa, Alabama, United States; Arkansas Tech University, Russellville, Arkansas, United States

## Abstract

With recent passage of the One Big Beautiful Bill Act (OBBBA), Medicaid changes are likely to impact older adults. One concern is that hospitals that depend on Medicaid revenue from higher Medicaid patient volumes may be at greater risk of closure. Alabama serves as an example of a state that did not expand Medicaid and provided limited aid to struggling hospitals. Even before the passage of the bill, a majority of Alabama’s hospitals were struggling partially due to a Medicare wage index calculation that underpaid hospitals (AHA, 2014). To better understand potential hospital access gaps in Alabama, we 1) coded media reports of at-risk hospitals in Alabama and hospitals who had reduced services/ converting to a rural emergency hospital (REH) 2) used the percent of Medicaid enrollment/eligibility by county in 2024. We then conducted GIS analyses to map the hospitals that may be at risk of closing or reducing services while examining Medicaid eligibility rates by county in Alabama. GIS analyses results revealed more at-risk hospitals in areas with higher Medicaid enrollment. Health access gaps were most pronounced in the lower half of the state, westernmost side of Alabama, and a pocket of 3 counties in Northeast Alabama. One hospital converted to an REH losing its geriatric psychiatry unit. While conversion to a REH may result in saving a hospital from closure and access to a nearby ER, the result is hardship for older adults depending on nearby inpatient services. Potential solutions to offer quality health care options are discussed.

